# Yield, Antioxidant Components, Oil Content, and Composition of Onion Seeds Are Influenced by Planting Time and Density

**DOI:** 10.3390/plants8080293

**Published:** 2019-08-20

**Authors:** Carmine Amalfitano, Nadezhda A. Golubkina, Laura Del Vacchio, Giuseppe Russo, Mario Cannoniero, Silvano Somma, Giuseppe Morano, Antonio Cuciniello, Gianluca Caruso

**Affiliations:** 1 Department of Agricultural Sciences, University of Naples Federico II, 80055 Portici (Naples), Italy; 2 Federal Scientific Center of Vegetable Production, Selectsionnaya str. 14, VNIISSOK, Odintsovo District, Moscow 143072, Russia; 3 Green Company, 84084 Fisciano, Salerno, Italy; 4 Council for Agricultural Research and Economics (CREA)—Research Center for Cereal and Industrial Crops, 81100 Caserta, Italy

**Keywords:** *Allium cepa* L. ‘Ramata di Montoro’, coldstored bulbs, plant biometrical and growth indicators, seed production, fatty acids, proteins, polyphenols, selenium

## Abstract

Research was carried out on onion landrace (Ramata di Montoro) for seed production in southern Italy, with the aim to evaluate the effects on yield and quality of four bulb planting times in factorial combination with four densities, using a split plot design with three replicates. The number of flower stalks per plant, their height and diameter, and the inflorescence diameter decreased with the bulb planting delay and density increase. The highest plant leaf area and LAI (leaf area index), seed yield, number, and mean weight were recorded with the earliest planting time, with the lowest bulb density eliciting the highest plant leaf area but the lowest LAI and seed yield per hectare. The ratio between seeds and inflorescence weight, and seed germinability, decreased with the planting delay and density increase. Seed oil, protein, and antioxidant content (polyphenols and selenium) were highest with the last crop cycle. The polyunsaturated fatty acids, predominant in oil, increased with planting time delay, whereas the monounsaturated fatty acids decreased. Linoleic, oleic, and palmitic acid prevailed among polyunsaturated, monounsaturated, and saturated fatty acids, respectively. Planting from 20 December to 10 January with 3.3 cold-stored bulbs per m^2^ was the most effective combination in terms of seed yield per hectare, whereas seed oil content and quality were the best, with the last crop cycle starting on 21 February, independent of bulb density.

## 1. Introduction

Onion (*Allium cepa* L.) belongs to Liliaceae, and, as reported in the FAO (Food and Agriculture Organization) database, it is the second most cultivated vegetable in the world after the potato, with a total surface area of about 5.4 million hectares (ha) and 103 Mt production, mostly produced in Europe and Asia, with China and India being the major producing countries [1]. In Italy, the surface devoted to onion is about 12 thousand ha [2]. Moreover, there are several high quality landraces, among which ‘Ramata di Montoro’ took origin in the Irno valley plain, including the town of Montoro (Avellino province, Campania region, southern Italy) in the late 19th century [3]. Notably, Ramata di Montoro is a storage onion whose bulbs are consumed for their qualitative and aromatic characteristics [4,5]. 

Seed production represents the first crucial step along the onion chain. Notably, seed crop can be carried out in the field, starting from seeds, seedlings or bulbs [6]. Direct sowing or transplanting falls in mid-summer to early autumn, which is due to the plant vernalization requirement for the turning to the reproductive phase. The use of bulbs stored at 10–12 °C is the most widespread method, as it results in a shorter crop cycle as well as a higher and more uniform seed yield [6]. Bulb planting occurs from autumn to spring in Mediterranean areas or from late winter to early spring in northern regions, and, along with bulb density, it is among the major factors greatly affecting the growth and yield of onions [4], as well as seed yield and quality [7,8]. In particular, Ashagrie et al. [9] recorded in northern Ethiopia the significant effect of planting time on stalk diameter, seed yield, and germination rate, with the best results corresponding to the earliest planting of 25 October. In other research [10], the 10 cm spacing along the rows led to a higher yield per surface unit compared to 15, 25, and 30 cm, whereas the highest production per plant was recorded under the lowest plant density, ensuing 30 cm spacing between the rows.

In addition to the use as propagation material, onion seeds represent a valuable human diet integrator, thanks to their remarkable nutrient value [11]. Notably, they contain an appreciable oil content (22–26%) with a high percentage of linoleic acid (49–61%), followed by oleic and palmitic acid; proteins (16–26%); alcohols, acids, and esters; sulphur-containing compounds [12], contributing to glycemic control and oxidation phenomena reduction; steroidal saponins, acting in cholesterol, diabetes, and cancer control; free amino acids [13], and antioxidants [14]. In this respect, Dini et al. [14] found that onion seeds from the variety Tropeana contain 20.4% oil, 24.8% raw proteins, and an appreciable polyphenol concentration. 

Oils are an important component of the human diet, and their accumulation in seeds as well as their fatty acid composition is affected by environmental, crop, and genetic factors [15]. In investigations carried out by Parry et al. [16] on oil fatty acid composition, a high polyunsaturated fatty acids (PUFA)/ saturated fatty acids (SFA) ratio, the prevalence of linoleic acid (C18:2) with 64.0 to 65.2%, followed by oleic acid (C18:1) with 24.8 to 26.0%, and palmitic acid (C16:0) with 7.1% was detected. Notably, the high PUFA/SFA ratio helps prevent cardiovascular diseases and aterosclerosis [12]. However, fatty acids, mainly the mono- and polyunsaturated ones, are very sensitive to oxidation, which is limited by polyphenols as well as by selenium, which is another strong antioxidant present in onion seeds [17]. Selenium is a microelement able to protect the plants from different stress types, such as salinity, drought, ultraviolet radiation, heavy metals, and herbivore attacks [18]. According to Golubkina et al. [19], selenium plays an important role in protecting *Brassica chinensis* plants from bacterial diseases.

With the aim to investigate the dynamics of production and quality of onion seed-oriented crop systems, research was carried out to assess the interactions between bulb planting time and density on plant biometrical and growth indicators, as well as seed yield, oil content and composition, proteins, polyphenols, and selenium of the landrace Ramata di Montoro. 

## 2. Results and Discussion

### 2.1. Plant Biometrical and Growth Indices, Seed Yield, and Germinability

As regards the biometrical parameters (Table 1), both bulb planting time and density showed a significant effect on the number of stalks per plant, as well as their height and diameter, inflorescence (umbel) diameter, and leaf area index (LAI). 

The flower stalk number per plant decreased with the planting time delay, from 4 units under the 20 December planting to 2.7 with the 21 February planting. This trend is consistent with the reports of research carried out by El-Helaly and Karam [8] in Egypt. 

Moreover, the number of flower stalks decreased from 3.6 with two bulbs per m^2^ to three under the five bulbs per m^2^ density. The stalk height also showed a decreasing trend with the planting delay, with 13.6% reduction from the earliest to the latest planting time. Moreover, the bulb density increase from two to five per m^2^ caused the stalk height reduction from 112.7 to 105.9 cm. The flower stalk diameter decreased from 41.9 mm with the first planting to 35.5 mm under the last one. The increase from the lowest to the highest bulb density resulted in 10.4% reduction of the stalk diameter. Our research findings are consistent with those recorded in previous research [8,9,20].

The umbel diameter was also adversely related with the planting time, as the highest value was recorded under the earliest planting (81.2 mm) and the lowest was detected with the latest (63.8 mm). The highest umbel diameter was recorded with mid-November planting and the lowest in mid-January, as the earliest planting enhanced flower stalks growth under favorable climate conditions and provided the umbels with a higher nutrient supply, leading to flowering anticipation [20].

Mollah et al. [21] reported that, out of five planting dates ranging from 1 October to 30 November at 15-day intervals, the 15 November planting time led to the tallest plants and the highest number of stalks (4.65) and umbels per plant, as well as the highest umbel diameter, though it did not statistically differ from 30 October and 30 November plantings.

In the present research, the decreasing trend of flower stalk diameter with the bulb density raise (from 42.2 to 37.8 mm) confirmed previous findings [7].

The earliest planted crops showed a higher leaf apparatus and dry weight accumulation compared to that attained by the later crops, as can be observed in Table 2. This result is due to the longer growth time span of the crops planted on 20 December, which also accumulated a higher biomass amount. Similar results were obtained in previous research carried out on storage onion cultivar Ramata di Montoro for bulb production in southern Italy [4].

Leaf area and dry matter per plant decreased with the bulb density rise, from 935.3 cm^2^ per plant and 73.8 g, respectively, under two bulbs per m^2^ to 838.8 cm^2^ per plant and 59.6 g, respectively with five bulbs per m^2^. Conversely, leaf area and dry weight per m^2^ were enhanced by the bulb density increase, from 0.28 m^2^·m^−2^ and 147.6 g, respectively, under the lowest density to 0.63 m^2^·m^−2^ and 298 g, respectively, with the highest one. Contrastingly, Caruso et al. [4] did not record the significant effect of plant density on leaf area in bulb-oriented onions.

The two experimental factors, bulb ‘planting time’ and ‘density’, showed significant effects on yield components, ratio between seeds and umbel weight, and seed germinability (Table 3).

**Figure 1:**
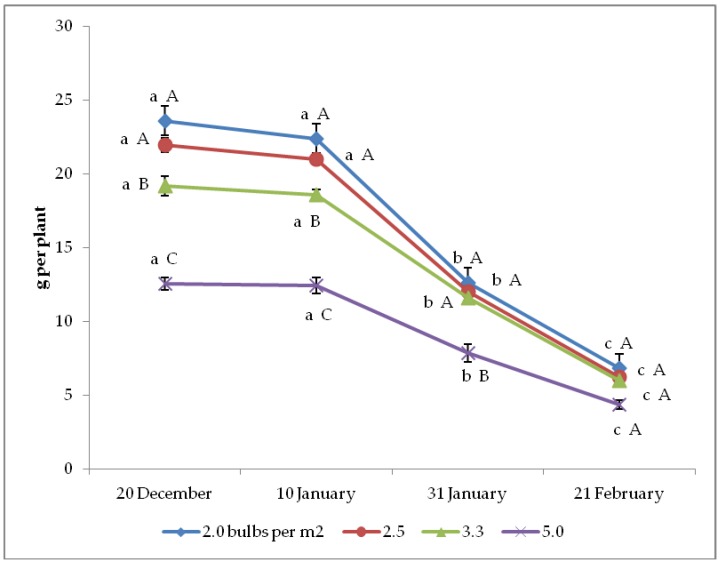
Interaction between bulb planting time and density on onion seed yield per plant (*n* = 3). Different letters mean significant difference in the comparison between planting times (lowercase letters) or between bulb densities (capital letters), according to the Duncan test at *p* ≤ 0.05. Standard deviation bars are shown per each value.

**Figure 2:**
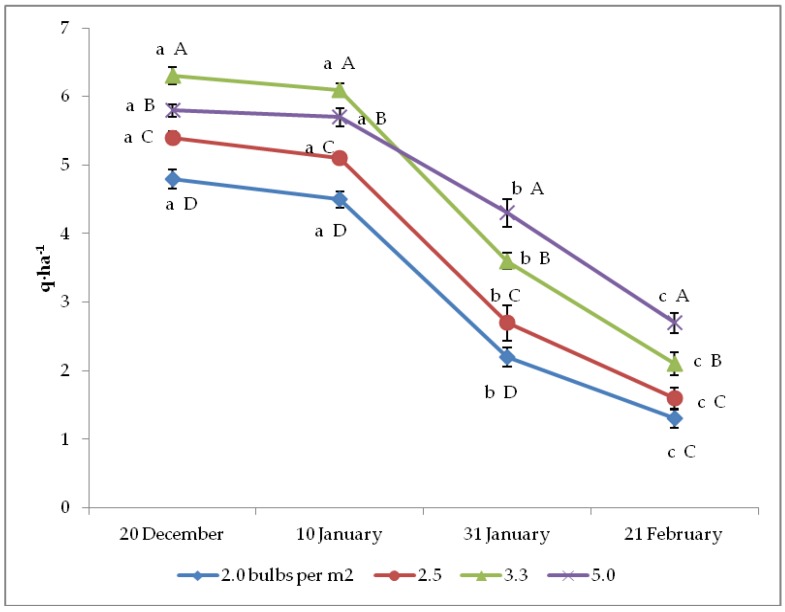
Interaction between bulb planting time and density on onion seed yield per ha (*n* = 3). Different letters mean significant difference in the comparison between planting times (lowercase letters) or between bulb densities (capital letters), according to the Duncan test at *p* ≤ 0.05. Standard deviation bars are shown per each value.

Both seed yield per plant and per hectare were significantly affected by the interaction between bulb planting time and density (Figure 1 and Figure 2). Indeed, in both cases the two earliest planting times (20 December and 10 January) showed the best results at any bulb density, whereas the latest planting (21 February) resulted in the lowest yield per plant. Similarly, the two lowest bulb densities (2.0 and 2.5 bulbs per m^2^) led to the highest yield per plant at any planting time, but they were not significantly different from 3.3 plants per m^2^ density in correspondence with the two latest plantings. Otherwise, yield per hectare showed the highest values, with 3.3 bulbs per m^2^ under the earliest planting time, and even with 5.0 bulbs per m^2^ upon the second planting, whereas it increased from 2.0 to 5.0 bulbs per m^2^ in the two latest crop cycles.

**Figure 3:**
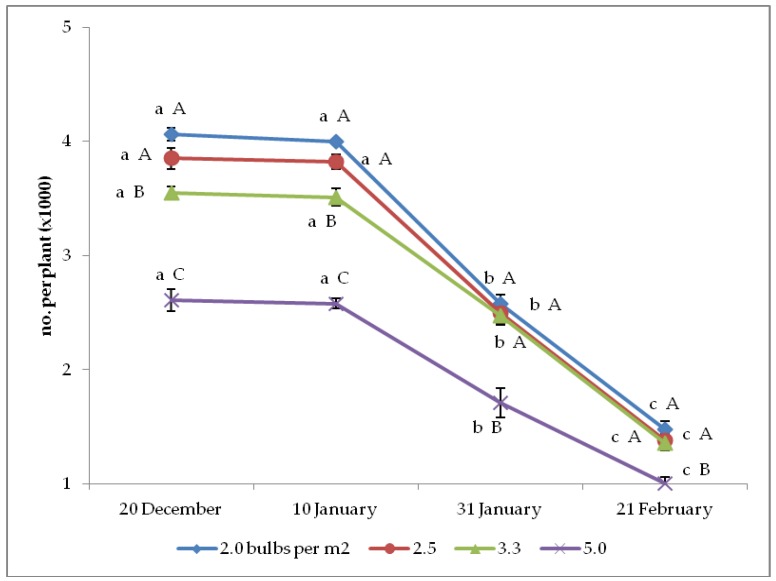
Interaction between bulb planting time and density on onion seed number per plant (*n* = 3). Different letters mean significant difference in the comparison between planting times (lowercase letters) or between bulb densities (capital letters), according to the Duncan test at *p* ≤ 0.05. Standard deviation bars are shown per each value.

**Figure 4:**
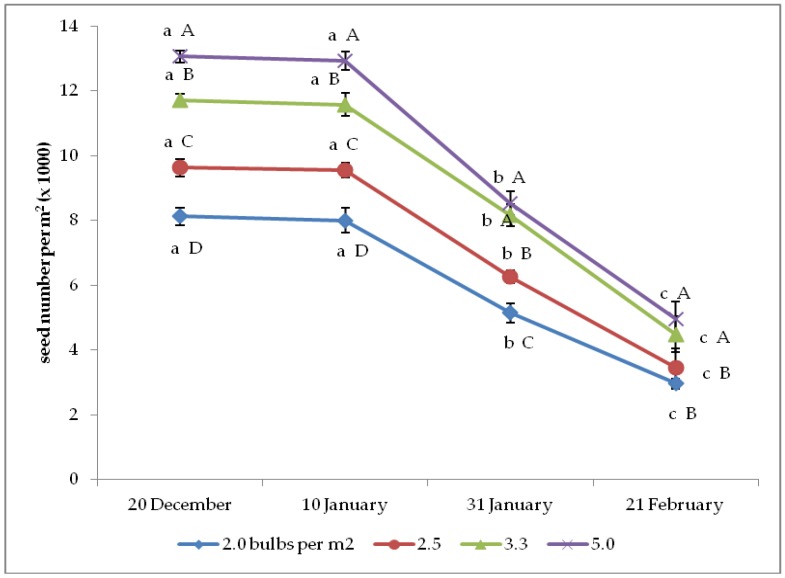
Interaction between bulb planting time and density on onion seed number per m^2^ (*n* = 3). Different letters mean significant difference in the comparison between planting times (lowercase letters) or between bulb densities (capital letters), according to the Duncan test at *p* ≤ 0.05. Standard deviation bars are shown per each value.

The interaction between bulb planting time and density was also significant on the seed number per plant and per square meter (Figure 3 and Figure 4). The former variable showed the same trends as described for yield per plant, whereas the trends of seed number per square meter only differed from the previous yield indicator because of the increase from 2.0 up to 5.0 bulbs per m^2^ under the two earliest plantings and up to 3.3 bulbs per m^2^ upon the two latest plantings.

**Figure 5:**
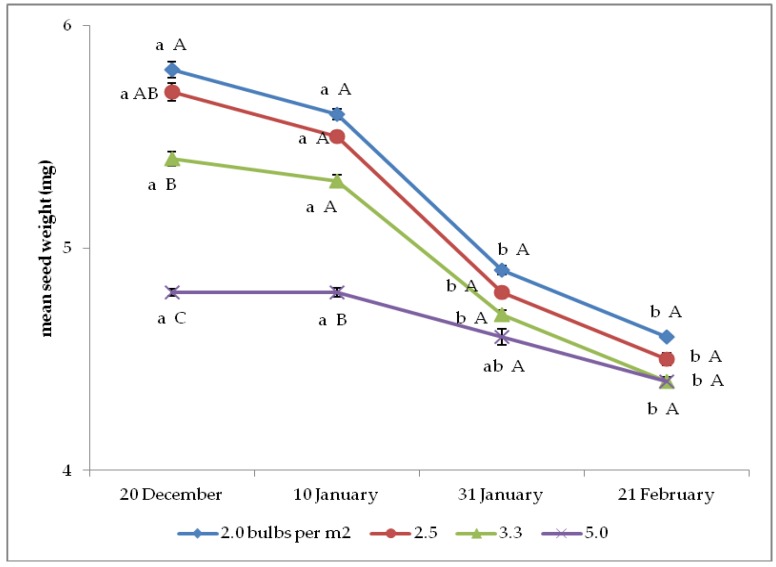
Interaction between bulb planting time and density on onion mean seed weight (*n* = 3). Different letters mean significant difference in the comparison between planting times (lowercase letters) or between bulb densities (capital letters), according to the Duncan test at *p* ≤ 0.05. Standard deviation bars are shown per each value.

The mean seed weight was significantly affected by the interaction between planting time and plant density (Figure 5). The two earliest planting times resulted in the highest values with 2.0 to 3.3 bulbs per m^2^, whereas they gave only better results than the latest planting correspondently to 5.0 bulbs per m^2^. Moreover, upon 20 December planting, the lowest bulb density led to higher mean seed weight than the two highest densities; under 10 January planting, only the 5.5 bulbs per m^2^ treatment was less effective than the other densities, and no significant difference between the treatments were recorded in correspondence with the two latest planting times.

Several correlations between the yield, growth, and biometrical variables examined were significant (Table 4). In particular, the seed yield per plant was positively correlated with both the seed number (r = 0.99 at *p* ≤ 0.01) and mean weight (r = 0.89 at *p* ≤ 0.01), whereas the seed yield per ha was not significantly correlated with the seed mean weight. Moreover, seed germinability showed significant positive correlations with all the mentioned parameters, except for LAI, which suggests the enhancement of this seed feature with the improvement of plant growth and productive conditions.

In previous research [22], the 10 cm spacing along the row was found more suitable both for obtaining higher onion seed yield and lower intensity of crop management labor in Sudanese environmental conditions, compared to 2.5 and 5.0 cm.

Mollah et al. [21] reported that, out of five planting dates ranging from 1 October to 30 November at 15-day intervals, the 15 November planting time resulted in the highest values of leaf area index (3.97) and seed yield (1.13 t·ha^−1^), both due to seed number per umbel (300) and mean seed weight (4.2 mg), as well as seed germination rate (84%); the lowest values were obtained with the two plantings practiced in the first half of October.

Khodadadi [23] in Iran found that 6 November led to taller plants and higher seed yield of onions compared to 5 March. Aminpour and Mortazavibak [24] reported that Texas Early Grano 502 cultivar in Isfahan showed the highest number of umbels and seed yield (1.4 t ha^−1^) when planted on 22 September. The latter date was found as the most appropriate for onion vegetative growth under the Karaj temperate climate, whereas the 21 November planting best fitted the Bangladesh tropical conditions [25].

El-Helaly and Karam [8] observed on cultivar Giza 20 grown in Egypt that the highest number of seed stalks, umbels, and seed yield per plant, as well as total seed yield, were associated to 15 November planting and the lowest to 15 January.

In other research [26], plant height, as well as the number of flowering stalks and of umbels per plant, decreased from 22 September to 5 November; however, seed yield was best affected by the planting dates ranging from 22 September to 6 October or from 21 October to 5 November, depending on the cultivar, and showed the same trend as the number of capsules per umbel and of seeds per capsule. Notably, yield increase resulted from the reduction of flowering stalk number and concurrent rise of umbel diameter, whereas the decrease recorded on 5 November was caused by adverse high temperature during July–August which were unfavorable for pollination, seed setting, and development [26]. In a different area [27], flower pollination decreases due to spring high temperatures and insecticide use which negatively affect seed setting and production. Khokhar [6] reported that the time from planting to inflorescence emergence and to floret opening increased linearly from the earliest planting date of 5 December (148 and 187 days, respectively) to the latest of 5 March (167 and 206 days, respectively); the highest seed yield was obtained under the earliest planting (5 December), when the conditions were more favorable for inflorescence initiation, with flowering occurring in early June and seed ripeness in mid-August. Mohamedali and Nouri [28] recorded the optimal planting time between mid-October to mid-November for onion seed yield, and the decrease of seed production upon later sowing was mainly caused by flower abortion increase.

The most effective temperature range for promoting the inflorescence primordia initiation is 9 °C to 13 °C [29]; however, cultivars differ in their optimal temperature requirements depending on the location to which they are adapted. Indeed, varieties from the northern or southern Russia have an optimal temperature range of 3 °C to 4 °C or of 9 °C to 10 °C, respectively. Though some authors [30] reported no significant effect of photoperiod on inflorescence initiation, in other research the time to this phase occurrence under inductive temperatures (9–13 °C) was reduced by increasing the photoperiod from 8 to 20 h per day in several cultivars [31,32,33]. In this respect, Brewster [32] reported the need of 86-day and 38-day exposure under 8 and 20 h photoperiod, respectively, in cultivar Rijnsburger; however, other varieties showed the requirement of over 14–16 h and, in cultivar Senshyu, the low sensitivity to photoperiod. In other investigations [6], the varieties showed different behavior, being inducted to inflorescence initiation under a short photoperiod (8 h d^−1^) at 11 °C to 13 °C, or under short to intermediate daylength (11 h d^−1^ to 14 h d^−1^) at 11 to 19 °C.

Following primordia initiation, a wider temperature range of 10 °C to 20 °C favors the emergence of inflorescence [34], which is fastest at 20 °C [35]. Over 20 °C, flower stalks fail to emerge [30], even upon the planting of sets with well-developed inflorescence initials [36]. Indeed, temperature exceeding this threshold halts inflorescence emergence directly when applied under short day conditions [30], and indirectly under long days as it promotes bulbing [37].

Once an inflorescence has been initiated, the rate of stalk elongation increases with rising temperature from 10 °C to 30 °C and a lengthening photoperiod to 14–16 h per day [33,35,38,39], both in over-wintered and spring-sown onion cultivars [40]. Contrastingly to each other, in a research study, Bertaud [35] found that the inflorescence emergence rate was faster under a 14 h photoperiod at 20 °C, whereas in other investigations 15 h at 10–16 °C was the most effective treatment [33].

Brewster [38] also reported that from 20 °C to 30 °C, both the growth of emerged flower stalks and the seed ripening show a 4–5 week anticipation compared with plants kept at 15 °C to 20 °C; however, under 16 h day length in plants with developed inflorescence initials, the inflorescence number is reduced, thus resulting in a larger proportion of shoots forming bulbs rather than inflorescences. Brewster [34] recorded the opening of florets 11 days earlier at 22 °C as compared to 16 °C, and Van Kampen [41] found that the temperature of 30 °C during the early stalk development can result in flower abortion because of assimilate competition with the bulbing process, though this is combined with the appropriate cultivar day length requirement.

The percentage of flower stalks with well-developed inflorescence initials and the floret number per umbel showed decreasing and increasing trends, respectively, upon increasing mean temperature from 14 °C to 23 °C and day length from 11 to 17 h per day [40,42], with the latter temperature and photoperiod trends, the time from planting to inflorescence initiation, spathe opening, flowering, and seed ripening decreases being a linear function of both climate parameters. Contrastingly, short days (10 h) can even halve the number of emerged inflorescences compared to plants grown under long days (14 to 18 h), also reducing flower stalk growth and flower number per umbel [29]; however, in some cultivars the number of plants with emerging inflorescences are increased by a 10 h photoperiod [36].

The ratio between seeds and inflorescence weight showed a decreasing trend with the planting time delay, with the highest value of 20% under the first planting being not significantly different from the second one, and the lowest being of 12.5% with the last crop cycle (Table 3). Moreover, this variable value decreased when the bulb density raised from two to five bulbs per m^2^ (from 17.5 to 16.8%, respectively).

As far as seed germinability is concerned (Table 3), the planting time delay caused significant decreases, as the highest value was recorded under the 20 December planting (98%) and the lowest with the latest planting performed on 21 February (74%).

Bulb density affected the seed germinability, which showed the highest value of 89% under the two bulbs per m^2^ treatment and the lowest of 82% with five bulbs per m^2^.

In previous investigations [43], the germination rate ranged between 80 and 90%. According to Maciel and coworkers [44], the genotype plays an important role on seed germinability in interaction with temperature, which is deemed crucial even by other authors [45]. Mollah et al. [21] reported that the 15 November planting time resulted in the highest seed germination rate (84%), whereas 1 and 16 October had the lowest.

### 2.2. Quality Indicators

#### 2.2.1. Oil Content and Fatty Acid Composition

As it can be observed in Table 5, onion Ramata di Montoro seeds showed an increasing oil percentage with the planting time delay, from 14.9% of the first planting to 16.5% of the last one.

From the gas-cromatographic analysis of fatty acid composition of onion Ramata di Montoro seed oil (Table 6), it was revealed that the polyunsaturated component was the most abundant (about 60%), followed by the monounsaturated (28–29%), and by the saturated (10–11%).

As for planting time (Table 6), planting delay led to saturated fatty acid increase from 9.2% to 11.5%, whereas the monounsaturated component showed a reduction from 32.7% to 25.4% and the polyunsaturated raised from 58.0% to 63.1%. The most abundant fatty acids were palmitic (6.8 to 8.5%), followed by stearic (1.6 to 2.0%), among the saturated; oleic (23.4 to 30.8%) among the monounsaturated, and linoleic (55.6 to 61.1%) among the polyunsaturated.

In previous research [17], Italian and Russian onion cultivars were tested, and higher oil percentages in Ramata di Montoro and Rossa di Tropea seeds were recorded in comparison to Russian cultivars (16.6% vs. 10.7 to 13.5%, respectively). Rossa di Tropea seeds showed an even higher oil occurrence (20.4%) in other investigations [14], whereas Yalcin and Kavuncuoglu [12] reported seed oil percentages ranging between 21.9 to 25.9% in 10 red Turkish varieties. Therefore, it can be inferred that red onions contain a higher oil percentage compared to lighter skin bulbs [46].

Similar seed fatty acid composition to that recorded in our study was reported in previous research [12,17]. Moreover, the values and ranking of fatty acids obtained in our investigation are similar to those reported by both Golubkina and coworkers [17] and Yalcin and Kavuncuoglu [12].

Moreover, Golubkina et al. [17] found a similarity between the fatty acid composition of onion seed oil and that of a cluster of natural oils, such as grapes, poppy, sunflower, and safflower. As for lipids, one of the most important issues concerning an oil rich with these compounds is the high oxidation sensitivity [46]. In this respect, the most susceptible fatty acid to oxidation is the oleic one, and this phenomenon is mainly caused by the high temperature in the Mediterranean summer season when the seed harvest occurs. In addition, the oleic acid synthesis causes the inhibition of linoleic acid biosynthesis [47].

In soybeans, early planting resulted in higher oil and oleic acid, both under single- and double-row growing, whereas late planting resulted in higher protein and linolenic acid, but lower oleic acid and oil concentrations [48]. In soybeans, significant correlations were recorded between seed size and content of fatty acids, i.e., positive with stearic and oleic, adverse with linoleic [49]. The ratio between saturated and unsaturated fatty acids in soybean oil is affected by extreme minimum daily temperatures during the seed fill period rather than by mean or maximum temperatures [50]. Both in cumin and chia seeds, fatty acid composition was greatly dependent on the environmental conditions [51,52].

Investigations carried out in different countries (Ethiopia, Bangladesh, Sudan) suggest the significant effects of planting time and spacing in interaction with environmental conditions on onion seed yield and quality [21,53]. Notably, research conducted in Sudan showed that smaller plant spacing does not have significant effect on onion seed quality, but it can reduce the ageing rate during storage. However, the 10 cm spacing along the row is recommended for obtaining higher onion seed quality in Sudanese environmental conditions, compared to 2.5 and 5.0 cm [22].

#### 2.2.2. Protein, Polyphenol, and Selenium Content

The protein percentage in onion Ramata di Montoro seeds increased with the planting time delay, from 19.7% to 22.0% (Table 5).

The polyphenol content in onion seeds increased with the planting time delay, from 2.14 mg·g^−1^ of fresh weight under the earliest planting to 2.40 mg·g^−1^ with the latest one (Table 5).

The selenium percentage increased from the first to the last crop cycle, ranging from 235 μg·kg^−1^ of fresh weight to 261 μg·kg^−1^, respectively (Table 5).

From the correlation analysis reported in Table 7, it arose that oil content, proteins, polyphenols, and selenium showed only significant positive correlations between each other and a negative correlation with the mean seed weight. The latter correlation suggests that the increase of seed dimensions results in the decrease of oil percentage as well as protein and antioxidant content, though it elicits higher seed yield per plant and germinability as inferred by the relevant positive correlations reported in Table 4.

Protein values recorded in our research are similar to those obtained by Golubkina and coworkers [16] in the Italian varieties Ramata di Montoro and Rossa di Tropea, and they fell in the range of values recorded in the Russian cultivars examined in the same research. Contrasting data were recorded in onion Rossa di Tropea seeds by Dini et al. [14], as the same authors found that red onion seeds are characterized by higher protein concentration than lighter skin bulb cultivars [11].

Polyphenol content detected in our trial are consistent with those reported in previous investigations [17]. In this respect, Žilić et al. [54] demonstrated that polyphenols play an important role in sunflower seed oil, by protecting it from oxidation during storage.

Selenium concentrations detected in our investigation are consistent with those detected by Golubkina and coworkers [17] in the onion Ramata di Montoro seeds. The same authors found a positive correlation between oil content and selenium concentration; notably, the latter plays an important antioxidant function, reducing PUFA peroxidantion. In fact, according to both Hartikainen and coworkers [55] and to Xue and coworkers [56], Se enhances the glutathione peroxidase activity or superoxide dismutase in plants, thus protecting the tocopherol from reduction processes.

Even in lettuce seeds produced under selenium supply, Xue et al. [56] demonstrated that this element prevents the lipids’ auto-oxidation, thus increasing the germination percentage. Moreover, Golubkina and coworkers [16] found a positive correlation between flavonoids and Se concentration in *Brassica juncea* [57], which was confirmed in research carried out on the effects of selenium fertilization in other species [58,59,60].

## 3. Materials and Methods

### 3.1. Experimental Protocol and Growth Conditions

**Figure 6:**
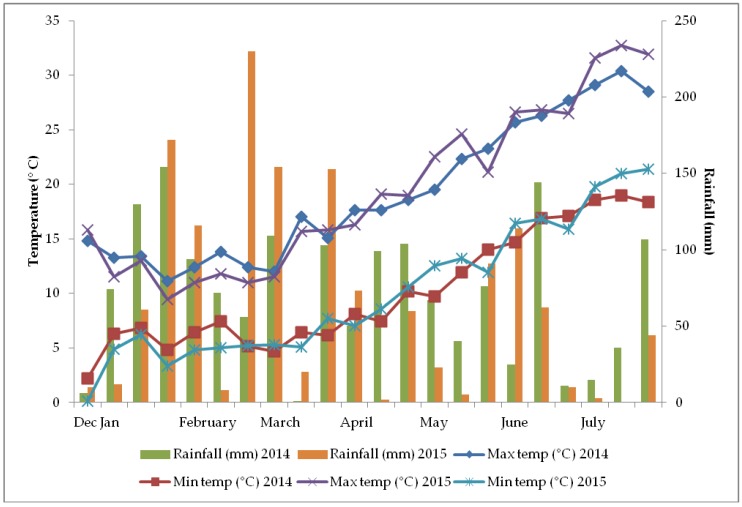
Trend of temperature and rainfall in Montoro (Avellino, southern Italy) in 2014 and 2015.

Research was carried out on the onion landrace Ramata di Montoro for seed production in open field, in Montoro (Avellino, southern Italy) in 2014 and 2015, on a sandy-silty soil with 54% sand, 27.3% silt, 18.8% clay, 2.5% organic matter, 0.3% nitrogen, 31 ppm P_2_O_5_, 376 ppm K_2_O, pH 6.5, and electrical conductivity (EC) 250 µS·cm^−1^. The trend of temperature and rainfall recorded in the research site is shown in Figure 6.

The experimental protocol was based on the factorial combination between four bulb planting times (20 December, 10 January, 31 January, 21 February) and four densities (2.0, 2.5, 3.3, 5.0 bulbs per m^2^); a split plot design was arranged with three replicates and the experimental unit had a 18 m^2^ surface area (4.5 × 4.0 m). The four bulb densities were achieved by spacing the rows 100 cm apart and by using the following distances between the bulbs along each row: 50, 40, 30, 20 cm, corresponding to 2.0, 2.5, 3.3, 5.0 bulbs per m^2^, respectively.

The bulbs used for the present research, having a 71 to 95 mm caliber, were harvested the previous year in July, ‘cured’ for two weeks, and stored at 5 °C for five months until the scheduled planting times.

The crops were preceded by cauliflower cultivation in both the research years and they were managed in compliance with the farming practices commonly used in the research area for the onion landrace Ramata di Montoro. Prior to bulb planting, ploughing was performed at 40 cm depth and hoeing at 15 cm. The fertilization pattern was in compliance with Khokhar [61] recommendations, supplying the plants with 70 kg·ha^−1^ of N (ammonium sulphate), 80 of P_2_O_5_ (mineral perphosphate) and 130 of K_2_O (potassium sulphate) before planting, and 130 kg·ha^−1^ of N and 140 of K_2_O (calcium nitrate and potassium nitrate) during the crop. Drip irrigation was activated when the soil available water capacity decreased to 80% and plant protection was performed with copper oxychloride application against rust.

### 3.2. General Analytical Methods

At the growth peak of leaf apparatus, leaf area was assessed using a Li-Cor3000 area meter (Li-Cor, Lincoln, NE, USA) on a three-plant sample per plot, and LAI (leaf area index) was also calculated. Moreover, just prior to seed ripening, the following determinations were performed: number of flower stalks per plant, as well as their height and diameter, and inflorescence diameter. Then, at crop cycle end, plant dry weight was assessed on a three-plant per plot sample. In the same phenological phase, i.e., on 2–6, 8–12, 14–16, and 17–19 July for the first, second, third, and fourth planting times, respectively, umbel harvest was performed in each plot in the early morning when seed humidity ranged between 45% and 65%, after which the seed-containing umbels were allowed to dry to 7% humidity in a rain-protected and naturally ventilated premise. Next, seeds were separated from umbels and the following determinations were done per each experimental treatment: weight of umbels and seeds per plant, mean weight of umbels and seeds, and ratio between seed and umbel weight. At the same time, a seed sample collected from each plot was transferred to the laboratory in order to assess the germinability in compliance with the official methods of seed determinations [62] and the quality attributes described below.

### 3.3. Qualitative Analyses

Fifty grams of seeds were randomly sampled in each plot, in order to perform the following chemical analyses: oil content and fatty acid composition, proteins, total polyphenols, and selenium.

Oil content was gravimetrically assessed through the seed extraction with n-esane (boiling point: 60 °C) for 6 h in a Soxhlet extractor, according to AOAC (Association of Official Analytical Chemists) [63].

Fatty acid composition was assessed through gas cromatography, according to AOCS (American Oil Chemists Society) [64]. A Perkin Elmer AutoSystem gas cromatograph, with Agilent J&W DB-Wax column (30 m × 0.25 mm, 0.25 μm), was used. PUFA and SFA have been used across the text to indicate polyunsaturated fatty acids and saturated fatty acids, respectively.

Total protein content (N × 6.25) was assessed by the Kjeldahl method [63].

Total polyphenol determination was performed as previously described [65]. In this respect, the seeds were finely ground and 10 mg of powder was extracted in 2420 mL Eppendorf metanol water solution (60%) for 4 days under discontinuous stirring through Tissue Lyser Qiagen equipped with stainless steel balls at 50 Hz. A limpid extract aliquot (40 µl), recovered through centrifugation (7000 rpm per 5′), underwent reaction with Folin–Ciocalteu colorimetric reagent and it was analyzed at 760 nm by a UV-VIS Perkin Elmer Lambda 25 spectrophotometer. Gallic acid was used to achieve the calibration line.

Selenium content was assessed by the microfluorimetric method, as reported by Alfthan [66].

### 3.4. Data Statistical Processing

Data were processed by three-way analysis of variance and mean separations were performed through the Duncan’s multiple range test, with reference to 0.05 probability level, using SPSS software version 21. Data expressed as percentage were subjected to angular transformation before processing. Correlations were performed between all the examined variables.

## 4. Conclusions

From research carried out in southern Italy on onion landrace Ramata di Montoro in the Montoro plain, which is its area of origin and cultivation since the 19th century, significant effects of bulb planting time and density on seed yield and quality arose. Indeed, the planting delay from beginning to late winter resulted in seed production and germinability decrease. However, seed quality in terms of oil, protein, and antioxidant (polyphenols and selenium) content was enhanced by postponing the crop cycle, thus starting plant growing under better light and temperature conditions.

Bulb density did not affect the chemical composition of Ramata di Montoro seeds, but its increase from two to five bulbs per m^2^ resulted in germinability percentage reduction, and it showed significant interaction with the planting time on seed yield.

From the present investigation, it can be inferred that onion landrace Ramata di Montoro crops started in the three earliest weeks of winter with 3.3 coldstored bulbs per m^2^ showed the highest seed yield per hectare, whereas seed oil content and quality were best affected by the latest planting (21 February) independently on bulb density, and seed germinability was negatively influenced by planting delay and density increase.

## Figures and Tables

**Table 1:** Mean values of onion biometrical parameters at harvest as affected by bulb planting time and density (*n* = 3 ± SD).

Source of Variance	Flower Stalks			Umbel Diameter (mm)
	No. Per Plant	Height (cm)	Diameter (mm)	
Year (Y)				
2014	3.1 ± 0.03	107.4 ± 0.99	39.1 ± 0.13	73.5 ± 0.74
2015	3.3 ± 0.02	111.1 ± 0.86	40.8 ± 0.23	76.1 ± 0.59
	n.s.	n.s.	n.s.	n.s.
Planting time (Pt)				
20 December	4.0 ± 0.07 ^a^	112.1 ± 1.96 ^a^	41.9 ± 0.74 ^a^	81.2 ± 1.39 ^a^
10 January	3.2 ± 0.07 ^b^	118.3 ± 2.33 ^a^	41.9 ± 0.83 ^a^	78.4 ± 1.50 ^a^
31 January	3.0 ± 0.04 ^b,c^	109.9 ± 1.39 ^a^	40.5 ± 0.53 ^a^	76.2 ± 0.89 ^a^
21 February	2.7 ± 0.10 ^c^	96.9 ± 3.56 ^b^	35.5 ± 1.23 ^b^	63.8 ± 2.37 ^b^
Bulb density per m^2^ (Bd)				
2.0	3.6 ± 0.04 ^a^	112.7 ± 1.37	42.2 ± 0.46 ^a^	76.8 ± 0.82
2.5	3.3 ± 0.04 ^a,b^	109.7 ± 1.45	39.9 ± 0.51 ^a,b^	75.0 ± 0.97
3.3	3.0 ± 0.04 ^b^	108.9 ± 1.15	39.9 ± 0.41 ^a,b^	74.3 ± 0.72
5.0	3.0 ± 0.04 ^b^	105.9 ± 1.34	37.8 ± 0.54 ^b^	73.4 ± 0.94
		n.s.		n.s.
Y × Pt	n.s.	n.s.	n.s.	n.s.
Y × Bd	n.s.	n.s.	n.s.	n.s.
Pt × Bd	n.s.	n.s.	n.s.	n.s.
Y × Pt × Bd	n.s.	n.s.	n.s.	n.s.

Within each column, n.s. = no statistically significant difference; means followed by different letters are significantly different according to the Duncan test at *p* ≤ 0.05. Mean values ± standard deviations have been reported for the main effects of the experimental factors.

**Table 2:** Mean values of onion growth indices as affected by bulb planting time and density (*n* = 3 ± SD).

Source of Variance	Leaf Area (cm^2^ Per Plant)	Leaf Area Index (LAI) (m^2^·m^−2^)	Dry Weight (g Per Plant)	Dry Weight (g Per m^2^)
Year (Y)				
2014	852.1 ± 12.3	0.27 ± 0.004	65.4 ± 0.49	203.3 ± 2.72
2015	888.0 ± 3.4	0.28 ± 0.002	68.3 ± 0.79	212.2 ± 2.87
	n.s.	n.s.	n.s.	n.s.
Planting time (Pt)				
20 December	1517.5 ± 23.2 ^a^	0.48 ± 0.008 ^a^	93.3 ± 1.54 ^a^	293.3 ± 5.65 ^a^
10 January	748.0 ± 13.1 ^b^	0.24 ± 0.004 ^b^	89.5 ± 1.26 ^a^	279.7 ± 5.96 ^a^
31 January	664.4 ± 11.5 ^b^	0.21 ± 0.004 ^b^	54.1 ± 0.82 ^b^	166.9 ± 2.91 ^b^
21 February	552.1 ± 24.3 ^c^	0.17 ± 0.007 ^c^	30.7 ± 1.51 ^c^	91.6 ± 4.48 ^c^
Bulb density per m^2^ (Bd)				
2.0	935.3 ± 8.2 ^a^	0.19 ± 0.002 ^d^	73.8 ± 0.75 ^a^	147.6 ± 1.51 ^d^
2.5	869.7 ± 11.6 ^a,b^	0.22 ± 0.003 ^c^	71.7 ± 0.93 ^a^	179.3 ± 2.32 ^c^
3.3	838.3 ± 10.2 ^b^	0.28 ± 0.003 ^b^	62.5 ± 0.92 ^b^	206.3 ± 4.01 ^b^
5.0	838.8 ± 13.0 ^b^	0.42 ± 0.007 ^a^	59.6 ± 1.07 ^b^	298.0 ± 5.33 ^a^
Y × Pt	n.s.	n.s.	n.s.	n.s.
Y × Bd	n.s.	n.s.	n.s.	n.s.
Pt × Bd	n.s.	n.s.	n.s.	n.s.
Y × Pt × Bd	n.s.	n.s.	n.s.	n.s.

Within each column, n.s. = no statistically significant difference; means followed by different letters are significantly different according to the Duncan test at *p* ≤ 0.05. Mean values ± standard deviations have been reported for the main effects of the experimental factors.

**Table 3:** Mean values of yield parameters and germinability of onion seeds as affected by bulb planting time and density *(n* = 3 ± SD).

Source of Variance	Yield Per Plant (g)	Yield Per ha (q)	Mean Seed Weight (mg)	Seed Number Per Plant	Seed Number Per m^2^	Seeds/Umbel Weight (%)	Seed Germinability (%)
Year (Y)							
2014	12.2 ± 0.13	3.8 ± 0.05	4.9 ± 0.01	2414 ± 21	7810 ± 103	16.7 ± 0.20	89.1 ± 0.45
2015	12.8 ± 0.10	4.0 ± 0.05	5.1 ± 0.01	2549 ± 14	8253 ± 124	17.3 ± 0.24	89.3 ± 0.57
	n.s.	n.s.	n.s.	n.s.	n.s.	n.s.	n.s.
Planting time (Pt)							
20 December	17.5 ± 0.43 ^a^	5.6 ± 0.13 ^a^	5.4 ± 0.03 ^a^	3275 ± 55 ^a^	10630 ± 194 ^a^	20.0 ± 0.89 ^a^	97.9 ± 1.32 ^a^
10 January	16.5 ± 0.23 ^a^	5.3 ± 0.11 ^a^	5.3 ± 0.04 ^a^	3138 ± 46 ^a^	10507 ± 110 ^a^	19.3 ± 0.78 ^a^	94.9 ± 1.23 ^a,b^
31 January	9.8 ± 0.18 ^b^	3.2 ± 0.06 ^b^	4.8 ± 0.02 ^b^	2159 ± 32 ^b^	7024 ± 110 ^b^	16.2 ± 0.25 ^b^	90.1 ± 0.96 ^b^
21 February	5.9 ± 0.26 ^c^	1.9 ± 0.08 ^c^	4.5 ± 0.04 ^b^	1343 ± 62 ^c^	3964 ± 226 ^c^	12.5 ± 0.57 ^c^	74.0 ± 2.76 ^c^
Bulb density per m^2^ (Bd)							
2.0	13.8 ± 0.26 ^a^	2.8 ± 0.05 ^d^	5.1 ± 0.13	2695 ± 28 ^a^	6060 ± 121 ^d^	17.5 ± 0.15 ^a^	92.9 ± 0.99 ^a^
2.5	13.3 ± 0.34 ^a^	3.3 ± 0.09 ^c^	5.0 ± 0.10	2647 ± 35 ^a^	7218 ± 82 ^c^	17.1 ± 0.46 ^a,b^	91.0 ± 1.12 ^a,b^
3.3	11.4 ± 0.17 ^b^	3.8 ± 0.07 ^b^	5.0 ± 0.09	2266 ± 32 ^b^	8979 ± 182 ^b^	17.0 ± 0.28 ^a,b^	88.0 ± 0.81 ^b,c^
5.0	11.2 ± 0.20 ^b^	5.6 ± 0.10 ^a^	4.8 ± 0.14	2318 ± 39 ^b^	9870 ± 232 ^a^	16.8 ± 0.13 ^b^	85.0 ± 0.84 ^c^
			n.s.				
Y × Pt	n.s.	n.s.	n.s.	n.s.	n.s.	n.s.	n.s.
Y × Bd	n.s.	n.s.	n.s.	n.s.	n.s.	n.s.	n.s.
Pt × Bd	n.s.	n.s.	n.s.	n.s.	n.s.	n.s.	n.s.
Y × Pt × Bd	n.s.	n.s.	n.s.	n.s.	n.s.	n.s.	n.s.

Within each column, n.s. no statistically significant difference; means followed by different letters are significantly different according to the Duncan test at *p* ≤ 0.05. Mean values ± standard deviations have been reported for the main effects of the experimental factors.

**Table 4:** Correlations between yield, growth, and biometrical parameters.

Parameter	Yield Per Plant	Yield Per ha	Mean Seed Weight	Seed Number Per Plant	Seed Number Per m^2^	Seeds/Umbel Weight	Seed Germinability	Flower Stalks Per Plant	Stalk Height	Stalk Diameter	Umbel Diameter	Leaf Area Per Plant	LAI
Mean seed weight	0.89 **	0.29 ^n.s.^	1.00	0.87 **	0.44 ^n.s.^	0.86 **	0.83 **	0.82 **	0.71 **	0.78 **	0.75 **	0.67 **	0.14 ^n.s.^
Seed number per plant	0.99 **	0.67 **	0.87 **	1.00	0.74 **	0.97 **	0.94 **	0.81 **	0.87 **	0.85 **	0.92 **	0.72 **	0.45 ^n.s.^
Seed number per m^2^	0.73 **	0.95 **	0.44 ^n.s.^	0.74 **	1.00	0.81 **	0.66 **	0.41 ^n.s.^	0.63**	0.50 *	0.72 **	0.55 *	0.73 **
Seeds/umbel weight	0.97 **	0.70 **	0.86 **	0.97 **	0.81 **	1.00	0.93 **	0.75 **	0.85 **	0.83 **	0.92 **	0.71 **	0.48 ^n.s.^
Seed germinability	0.93 **	0.56 *	0.83 **	0.94 **	0.66 *	0.93 **	1.00	0.80 **	0.92 **	0.95 **	0.98 **	0.67 **	0.35 ^n.s.^
Flower stalks per plant	0.83 **	0.39 ^n.s.^	0.82 **	0.81 **	0.41 ^n.s.^	0.75 **	0.80 **	1.00	0.58 *	0.74 **	0.77 **	0.92 **	0.47 ^n.s.^
Stalk height	0.85 **	0.54 *	0.71 **	0.87 **	0.63 **	0.85 **	0.92 **	0.58 *	1.00	0.94 **	0.91 **	0.38 ^n.s.^	0.17 ^n.s.^
Stalk diameter	0.84 **	0.40 ^n.s.^	0.78 **	0.85 **	0.50 *	0.83 **	0.95 **	0.74 **	0.94 **	1.00	0.93 **	0.53 *	0.17 ^n.s.^
Umber diameter	0.90 **	0.64 **	0.75 **	0.92 **	0.72 **	0.92 **	0.98 **	0.77 **	0.91 **	0.93 **	1.00	0.68 **	0.45 ^n.s.^
Leaf area per plant	0.74 **	0.55 *	0.67 **	0.72 **	0.55 *	0.71 **	0.67 **	0.92 **	0.38 ^n.s.^	0.53 *	0.68 **	1.00	0.73 **
LAI	0.45 ^n.s.^	0.83 **	0.14 ^n.s.^	0.45 ^n.s.^	0.73 **	0.48 ^n.s.^	0.35 ^n.s.^	0.47 ^n.s.^	0.17 ^n.s.^	0.17 ^n.s.^	0.45 ^n.s.^	0.73 **	1.00
Dry weight per plant	0.99 **	0.66 **	0.89 **	0.99 **	0.75 **	0.71 **	0.93 **	0.81 **	0.87 **	0.85 **	0.90 **	0.72 **	0.44 ^n.s.^
Dry weight per m^2^	0.66 **	0.98 **	0.29 ^n.s.^	0.68 **	0.96 **	0.71 **	0.57 *	0.39 ^n.s.^	0.55 *	0.41 ^n.s.^	0.65 **	0.54 *	0.81 **

^n.s.^ not statistically significant; * statistically significant at *p* ≤ 0.05; ** statistically significant at *p* ≤ 0.01.

**Table 5:** Mean values of oil, protein, and antioxidants content of onion seeds as affected by bulb planting time and density (*n* = 3 ± SD).

Source of Variance	Oil Content (%)	Proteins (%)	Polyphenols (mg·g^−1^ f.w.)	Selenium (µg·kg^−1^ f.w.)
Year (Y)				
2014	15.5 ± 0.13	20.6 ± 0.25	2.25 ± 0.04	243 ± 4.5
2015	15.9 ± 0.20	21.0 ± 0.43	2.30 ± 0.02	250 ± 1.5
	n.s.	n.s.	n.s.	n.s.
Planting time (Pt)				
20 December	14.9 ± 0.44 ^c^	19.7 ± 0.78 ^c^	2.14 ± 0.09 ^c^	235 ± 8.0 ^b^
10 January	15.3 ± 0.46 ^b,c^	20.4 ± 0.77 ^b,c^	2.22 ± 0.07 ^b,c^	243 ± 8.2 ^b^
31 January	16.0 ± 0.37 ^a,b^	21.3 ± 0.42 ^a,b^	2.35 ± 0.04 ^a,b^	248 ± 4.5 ^a,b^
21 February	16.5 ± 0.62 ^a^	22.0 ± 0.70 ^a^	2.40 ± 0.08 ^a^	261 ± 8.2 ^a^
Plant density per m^2^ (Bd)				
2.0	15.5 ± 0.47	21.1 ± 0.34	2.25 ± 0.04	245 ± 3.9
2.5	15.8 ± 0.40	20.6 ± 0.54	2.34 ± 0.04	242 ± 4.3
3.3	15.6 ± 0.33	20.9 ± 0.51	2.24 ± 0.05	253 ± 5.2
5.0	15.8 ± 0.45	20.8 ± 0.57	2.28 ± 0.07	247 ± 6.0
	n.s.	n.s.	n.s.	n.s.
Y × Pt	n.s.	n.s.	n.s.	n.s.
Y × Bd	n.s.	n.s.	n.s.	n.s.
Pt × Bd	n.s.	n.s.	n.s.	n.s.
Y × Pt × Bd	n.s.	n.s.	n.s.	n.s.

Within each column, n.s. not statistically significant; means followed by different letters are significantly different according to the Duncan test at *p* ≤ 0.05. Mean values ± standard deviations have been reported for the main effects of the experimental factors.

**Table 6:** Fatty acids composition of onion seeds as affected by bulb planting time (*n* = 3 ± SD).

Fatty Acid		20 December	10 January	31 January	21 February	
Saturated						
Lauric	12:0	0.03 ± 0.004	0.03 ± 0.004	0.02 ± 0.002	0.02 ± 0.002	n.s.
Miristic	14:0	0.13 ± 0.02 ^b^	0.13 ± 0.02 ^b^	0.14 ± 0.02 ^a,b^	0.16 ± 0.02 ^a^	
Pentadecanoic	15:0	0.05 ± 0.01 ^b^	0.06 ± 0.02 ^a,b^	0.06 ± 0.01 ^a,b^	0.07 ± 0.01 ^a^	
Pentadecenic	15:1	0.02 ± 0.003	0.02 ± 0.003	0.02 ± 0.001	0.03 ± 0.003	n.s.
Iso-esadecanoic	16:0 i	0.02 ± 0.002	0.02 ± 0.002	0.02 ± 0.002	0.02 ± 0.002	n.s.
Palmitic	16:0	6.84 ± 0.41 ^c^	7.67 ± 0.40 ^b^	8.10 ± 0.38 ^a,b^	8.48 ± 0.35 ^a^	
Margaric	17:0	0.04 ± 0.006	0.05 ± 0.004	0.05 ± 0.006	0.05 ± 0.004	n.s.
Stearic	18:0	1.57 ± 0.12 ^b^	1.78 ± 0.17 ^a,b^	2.02 ± 0.24 ^a^	1.97 ± 0.21 ^a^	
Iso-ottadecanoic	18:0 i	0.14 ± 0.012 ^a^	0.13 ± 0.006 ^a,b^	0.12 ± 0.010 ^b^	0.09 ± 0.004 ^c^	
Arachic	20:0	0.22 ± 0.03 ^c^	0.30 ± 0.03 ^b^	0.38 ± 0.03 ^a^	0.40 ± 0.04 ^a^	
Beeric	22:0	0.14 ± 0.01 ^a^	0.13 ± 0.01 ^a,b^	0.13 ± 0.01 ^a,b^	0.12 ± 0.01 ^b^	
Lignoceric	24:0	0.04 ± 0.004	0.05 ± 0.004	0.05 ± 0.004	0.04 ± 0.003	n.s.
Total		9.24 ± 0.62 ^c^	10.37 ± 0.64 ^b^	11.11 ± 0.47 ^a,b^	11.45 ± 0.51 ^a^	
Monounsaturated						
Sapienic	16:1 6-*cis*	0.07 ± 0.01	0.08 ± 0.01	0.08 ± 0.01	0.08 ± 0.01	n.s.
Palmitoleic	16:1 9-*cis*	0.15 ± 0.02 ^c^	0.17 ± 0.02 ^b,c^	0.18 ± 0.02 ^a,b^	0.20 ± 0.02 ^a^	
Eptadecenoic	17:1	0.03 ± 0.003	0.04 ± 0.003	0.04 ± 0.003	0.04 ± 0.003	n.s.
Oleic	18:1 9-*cis*	30.8 ± 1.58 ^a^	27.7 ± 1.71 ^b^	25.8 ± 1.65 ^b,c^	23.4 ± 2.23 ^c^	
Vaccenic	18:1 11-*trans*	1.28 ± 0.12	1.30 ± 0.12	1.29 ± 0.10	1.30 ± 0.10	n.s.
Gondoic	20:1 11-*cis*	0.41 ± 0.10	0.44 ± 0.06	0.43 ± 0.08	0.42 ± 0.10	n.s.
Total		32.74 ± 1.53 ^a^	29.76 ± 1.70 ^b^	27.82 ± 1.70 ^b,c^	25.44 ± 2.12 ^c^	
Polyunsaturated						
Iso-ottadecadienoic	18:2 i	0.07 ± 0.01	0.08 ± 0.01	0.07 ± 0.01	0.07 ± 0.02	n.s.
Linoleic	18:2	55.6 ± 2.48 ^b^	57.5 ± 2.51 ^a,b^	59.0 ± 2.86 ^a,b^	61.1 ± 2.62 ^a^	
α-linolenic	18:3	0.12 ± 0.01	0.11 ± 0.01	0.10 ± 0.02	0.12 ± 0.02	n.s.
γ-linolenic	18:3	0.03 ± 0.01	0.04 ± 0.01	0.02 ± 0.01	0.03 ± 0.01	n.s.
Punicic (isomers sum)	∑ 18:3	1.94 ± 0.15 ^a^	1.84 ± 0.13 ^a^	1.54 ± 0.14 ^b^	1.50 ± 0.16 ^b^	
Eicosatrienoic	20:3	0.03 ± 0.006 ^a^	0.03 ± 0.006 ^a^	0.01 ± 0.005 ^b^	0.01 ± 0.005 ^b^	
Arachidonic	20:4	0.25 ± 0.02	0.27 ± 0.03	0.29 ± 0.02	0.30 ± 0.02	n.s.
Total		58.02 ± 2.63 ^b^	59.87 ± 2.37 ^a,b^	61.07 ± 2.72 ^a,b^	63.11 ± 2.54 ^a^	

Along each line: n.s. not statistically significant; means followed by different letters are significantly different according to the Duncan test at *p* ≤ 0.05. Mean values ± standard deviations have been reported for the main effects of the experimental factors.

**Table 7:** Correlations between quality and yield, growth and biometrical parameters.

Parameter	Oil Content	Proteins	Polyphenols	Selenium
Yield per plant	−0.28 ^n.s.^	−0.28 ^n.s.^	−0.28 ^n.s.^	−0.28 ^n.s.^
Yield per ha	0.08 ^n.s.^	0.03 ^n.s.^	0.03 ^n.s.^	0.06 ^n.s.^
Seed number per plant	−0.26 ^n.s.^	−0.27 ^n.s.^	−0.26 ^n.s.^	−0.27 ^n.s.^
Seed number per m^2^	−0.11 ^n.s.^	−0.14 ^n.s.^	0.15 ^n.s.^	−0.09 ^n.s.^
Mean seed weight	−0.52 *	−0.50 *	−0.51 *	−0.51 *
Seeds/umbel weight	−0.36 ^n.s.^	−0.36 ^n.s.^	−0.37 ^n.s.^	−0.36 ^n.s.^
Seed germinability	−0.19 ^n.s.^	−0.18 ^n.s.^	−0.18 ^n.s.^	−0.20 ^n.s.^
Flower stalks per plant	−0.20 ^n.s.^	−0.19 ^n.s.^	−0.20 ^n.s.^	−0.21 ^n.s.^
Stalk height	−0.06 ^n.s.^	−0.05 ^n.s.^	−0.06 ^n.s.^	−0.06 ^n.s.^
Stalk diameter	−0.07 ^n.s.^	−0.04 ^n.s.^	−0.07 ^n.s.^	−0.07 ^n.s.^
Umber diameter	−0.13 ^n.s.^	−0.13 ^n.s.^	−0.13 ^n.s.^	−0.14 ^n.s.^
Leaf area per plant	−0.23 ^n.s.^	−0.24 ^n.s.^	−0.26 ^n.s.^	−0.24 ^n.s.^
LAI	0.05 ^n.s.^	0.01 ^n.s.^	−0.01 ^n.s.^	0.04 ^n.s.^
Dry weight per plant	−0.28 ^n.s.^	−0.28 ^n.s.^	−0.28 ^n.s.^	−0.28 ^n.s.^
Dry weight per m^2^	0.07 ^n.s.^	0.03 ^n.s.^	0.03 ^n.s.^	0.06 ^n.s.^
Oil content	1.00	0.99 **	0.99 **	0.99 **
Proteins	0.99 **	1.00	0.98 **	0.99 **
Polyphenols	0.99 **	0.98 **	1.00	0.97 **
Selenium	0.99 **	0.99 **	0.97 **	1.00

^n.s.^ not statistically significant; * statistically significant at *p* ≤ 0.05; ** statistically at *p* ≤ 0.01.
